# Epidemic Cholera

**Published:** 1849-02

**Authors:** 


*Epidemic Cholera.*—The epidemic which we announced in our last month's publication as having invaded this country, has continued to rage with great virulence in Edinburgh, where the number of cases now amounts to between seven and eight hundred (including Leith), of which nearly a half have already proved fatal, about 230 remaining under treatment. A severe epidemic broke out at Loanhead (a village six miles to the south of Edinburgh), and many cases have also occurred at Lasswade, Gilmerton, Portobello, Cockpen, and other villages of the neighborhood. Several cases have been reported in Glasgow, mostly in very crowded and miserable localities: and the disease is also reported to exist in Dumfries as well as in Falkirk, a town whose sanitary condition is far from satisfactory. In England the number of cases has not approached that of the northern division of the kingdom; and, although in London the number has been considerable, yet, relatively to the population, it is very small compared with Edinburgh. On the week ending October 4th, 65 deaths were recorded in the Registrar-

General's returns from cholera in London; on November 11th, 62; on November 18th, 54. The epidemic appears, therefore, on the whole, to be decreasing in violence in London. Everywhere the disease appears to have been almost confined to the most wretched and miserable of the population; a few trivial exceptions only to this rule having occurred in London and Edinburgh. The mortality has been everywhere nearly the same,—viz., about two-thirds of the recorded cases which have been brought to a termination. It is probable, however, that no trustworthy returns of the whole cases in any place have been yet published.

On the continent it has declined in a great measure at St. Petersburg, Berlin, and Hamburg, where, however, cases still occur. On the other hand, Rotterdam has been attacked with great severity since the 1st of October, and Dantzic, from a similar date, has suffered under a still more severe visitation. It is stated that in the small town of Gartz, in the district of Stettin, there has been the extraordinary mortality of 102 persons out of a population of 700.

A considerable number of cases of a choleroid disease prevailed in the beginning of the month in Dunkirk; and a few have occurred in the neighboring villages, and in Calais. M. Magendie was sent by the Academy of Sciences to investigate this alleged ingress of the disease into France; and reported the cases not to be true epidemic cholera. If we may trust the report of a discussion in the *Sociétié de Médecine Prutique*, several cases having the strongest resemblance to the disease appear to have occurred in Paris; but there seems to be, on the part of the French physicians, a strong disposition to doubt or deny its presence.

The General Board of Health has displayed considerable activity, but not all in the right direction. The truth is, that it has been hard beset. Composed, as it is in great part, of non-medical members, and forced to act in an emergency, the single medical head connected with it, has not been able to save it from being thrown upon the suggestions and recommendations of busy-bodies; the effect of which was the framing of a document full of inconsistency, and founded on most imperfect knowledge of the real views of the profession. The College of

Physicians of Edinburgh was the first to disclaim the views of the Board of Health, as we noticed in our last number; and this was followed by a most wise and temperate document on the part of the London College of Physicians, recommending hospitals and houses of refuge, and showing the absurdity of the attempt to dictate particular dietetic systems to persons who may be very dependent on many of the forbidden articles. Nearly the whole medical press of the country having likewise joined in tolerable unanimity on this subject, the General Board has been induced to remodel its instructions; and we have now before us a set of additional directions for England and another for Scotland, in which particular orders are given for the construction of cholera hospitals and houses of refuge, and for the employment, where necessary, of additional medical assistance.

We are happy to find that all the medical authorities have agreed to consider the question of contagion an open one, and have concurred expressing sentiments of a moderate character on this subject, closely corresponding with those which we endeavored to impress upon our readers in the last number of the Journal. We hope that in all hospitals and public institutions, facts relating to the propagation of cholera will be scrupulously and carefully recorded. Reports should be drawn up of the *whole* of the nurses and other persons exposed to contagion in such institutions, with the view of ascertaining the proportion in which they are affected with the disease. We believe that a large body of returns of this kind would be of more essential service than any other species of evidence on the subject of contagion.

A committee of the Edinburgh College of Physicians and Surgeons has been appointed, to procure evidence in relation to the present epidemic of cholera in Edinburgh and Scotland. Circulars have been issued to all the medical practitioners in Edinburgh, and the adjoining villages which have been invaded with cholera, enclosing blank schedules, to be filled up with information in a short form, in regard to any cases which may occur. We take this opportunity of impressing upon medical men the importance sf attending to this point, as the labors of the committee can only be rendered available for scientific purposes, by the coöperation of a large number of persons; and the trouble imposed on them by filling up the schedules is, from the simplicity with which these are drawn up, extremely trifling.

The most remarkable circumstance as to the treatment of cholera, arising out of the present epidemic, is the employment of the inhalation of chloroform in ten cases successively, in Peckham Asylum, without a single fatal result. This favorable experience has not been borne out in Edinburgh, where this remedy has been very assiduously tried in the Cholera Hospital without the least apparent good effect. The weekly and daily journals are full of remedies, but few of these rest on any large experience.—*Monthly Journal.*

